# Supplementary material for: ‘More than just numbers on a page?’ A qualitative exploration of the use of data collection and feedback in youth mental health services
Source: PLoS One. 2022 Jul 20;17(7):e0271023. doi: 10.1371/journal.pone.0271023 (PMC9299353; doi:10.1371/journal.pone.0271023)
Supplement: S1 Appendix — (PDF) [file pone.0271023.s001.pdf]

S1 Appendix.

Data collection and feedback in Youth mental health: Interview topic guide

Introduction

Thanks again for agreeing to take part in this research project, which forms part of my Master of Public Health. Today we're going to explore your experience of monitoring and evaluation in youth mental healthcare. We'll discuss what we mean by these terms in a minute.

We'll be interviewing about 40 people. The findings of the project may help inform the ways in which we can provide more support to PHNs and youth mental health services with monitoring and evaluation.

I'll ask you questions about your experience of being involved in monitoring and evaluation, who's responsible for different activities, and I'll also ask questions designed to identify barriers and enablers to using monitoring and evaluation information to inform service decisions.

We're interested in your personal experiences. There are no right or wrong answers. Whatever comes to mind is fine.

The interview will be audio-recorded and sent securely to a transcription service. To maintain your anonymity, you'll be assigned a participant number, which we'll use when using any quotes in the final report.

Define monitoring and evaluation

We're calling on the Department of Health's guidance for PHNs to explain what we mean by monitoring and evaluation.

When we talk about monitoring we're referring to the ongoing collection and analysis of service-related information, such as what service activities are being delivered; how services are being delivered; the extent to which outcomes are being achieved; compliance with contractual obligations; and the health of the relationship between the PHN and service provider.

Evaluation is a periodic activity which builds on monitoring information to enable PHNs to make judgments about the overall quality of the services they have commissioned. It can help PHNs and commissioned services to identify what has worked well and what has not, reasons for success or failure, and learning from both.

Do you have any questions before we start? Are you happy for me to turn on the recorder now? [TURN ON RECORDER]

That's me recording now, can you confirm for the recording that you're happy with this being recorded?

General (including knowledge)

S1 Appendix.

- Are you aware of what monitoring and evaluation activities are ongoing within your service/commissioned youth mental health services?
- What kinds of monitoring and evaluation information do you collect? (Knowledge)
- How were decisions made about what information you collect for monitoring and evaluation?
- How is the information collected for monitoring and evaluation being used? (Knowledge)
- How would you like to use the information? (Knowledge)
- What information do you find most useful? (Knowledge)

Actors and actions

- How is the information collected in the service, who is responsible for collecting it, and when does it happen?
- Who is responsible for data analysis and when does this happen?
- How is information interpreted, who is responsible and when does it happen?
- How is information fed back to the service, who does it, and when does it happen?
- How is information acted upon, who is responsible for making decisions about how it will be acted on, and when does it happen?

Social/professional role and identity

- Thinking about those monitoring and evaluation activities we've just spoken about. What responsibilities do you have in relation to monitoring and evaluation?
    - Which activities feature most in your day to day activities? Or which of those activites are you most familiar with or have most experience with?

NOTE: Remind participant to think about that particular activity in relation to the rest of the questions and tell them that they will be able to talk more generally toward the end of the interview.

Skills

- What skills do you think you need to be able to use monitoring and evaluation information?
- Do you have any training needs around using monitoring and evaluation information?

Memory, attention and decision processes

- Is using monitoring and evaluation information something you would normally do?
    - Do you rely on something to remind you?
    - Have you ever forgotten?

Behavioural regulation

- Are there any procedures, systems or ways of working that would make it easier for you to use monitoring and evaluation information?

Beliefs about capabilities

- How confident are you in using monitoring and evaluation information?
- What difficulties have you encountered when trying to use monitoring and evaluation information?
    - What would have helped you to overcome these problems?

Emotion

S1 Appendix.

- When you're using monitoring and evaluation information – do you ever feel worried/concerned/stressed or good about it?
    - Do you think this impacts on how you use the information?

Beliefs about consequences

- What do you think are the benefits and downsides of using monitoring and evaluation information?
    - Are there benefits and downsides to <u>not</u> use monitoring and evaluation information?
    - Are there times when the downsides outweigh the benefits?

Intentions

- How important is it for you to be making use of monitoring and evaluation information?

Goals

- What do you hope to achieve by using monitoring and evaluation?
- Does it conflict with any other priorities you have/ things you want to achieve?

Reinforcement

- Is there anything that encourages or discourages you to use monitoring and evaluation information?
    - e.g.incentives/rewards, or sanctions for not using ME information

Environmental context and resources

- Are there aspects of your organisation that make it easier or harder to make use of monitoring and evaluation information?
    - e.g. time, workload, competing tasks, staff, skills, equipment/software, organisational culture?
- Are there aspects related to your PHN/commissioned youth mental health services that make it easier or harder to use monitoring and evaluation information?
    - What could they do to help you improve use of information ?

Social influences

- In what ways do any of the following people and organisations influence your use of monitoring and evaluation information?
    - colleagues in your organisation
    - <u>for services</u>: other youth mental health services
    - <u>for PHNs:</u> other PHNs or the Federal Government
    - young people and families
    - any other organisations you can think of

Optimism

- How optimistic are you about using monitoring and evaluation information in the future?

Wrap up question

That's all the questions I had planned to ask you. Is there something that you feel is important about using monitoring and evaluation information that we haven't already covered?

S1 Appendix.

[TURN OFF RECORDER]

Thank you

- Thanks for taking part. I will send you a summary of the findings once the project is complete. In the meantime, if you have any questions, feel free to contact me anytime.
