# Supplementary material for: ‘More than just numbers on a page?’ A qualitative exploration of the use of data collection and feedback in youth mental health services
Source: PLoS One. 2022 Jul 20;17(7):e0271023. doi: 10.1371/journal.pone.0271023 (PMC9299353; doi:10.1371/journal.pone.0271023)
Supplement: S2 Appendix — (PDF) [file pone.0271023.s002.pdf]

S2 Appendix. Supp. Results Table

Summary table of relevant domains, specific beliefs, reason for relevance and illustrative quotations				
**Domains of the TDF**	**Specific beliefs**	**Frequency (utterances/ participants)**	**Criteria met**	**Illustrative quotes**
**Healthcare commissioners**				
Skills	You need to be able to build relationships with other organisations	35/14	*Evidence of strong beliefs impacting on behaviour, belief shared with youth mental health services*	*I think it's the relationships that we have with our providers, that enables I think the honesty and the transparency to be able to have these discussions. (Participant 2)*
	You need a good understanding of the youth mental health service context	17/12	*Evidence of strong beliefs impacting on behaviour, belief shared with youth mental health services*	*…it's important to be able to understand the context as well. So that's why I think it's much more valuable for staff that have more of a relationship with providers to be looking at data because we have a better idea of the context around what that - yeah, why the data is the way it is. (Participant 13)*
	You need to be data literate	21/12	*Evidence of strong beliefs impacting on behaviour, belief shared with youth mental health services*	*You need to be able to dissect what data actually means. I'm not qualified in any data analysis. You definitely need to have some element of understanding it, because otherwise it's just numbers and graphs. (Participant 40)*
	You need to be able to empathise with YMH service staff	19/10	*Evidence of strong beliefs impacting on behaviour, belief shared with youth mental health services*	*Having some communication skills and a bit of empathy goes a long way too for what it must be like to be out there being a service provider, seeing a bunch of people who are really sick, too sick for your service but having nowhere else for them to go so you just do the best you can with what you got. (Participant 17)*
	You need to be inquisitive and open minded	11/5	*Evidence of strong beliefs impacting on behaviour, belief shared with youth mental health services*	*…we would expect with some of our services to see more incident reports than we do. Particularly the lower severity incidents because of - by virtue of the cohort of folks that they support. Having to have those conversations with that curiosity lens around, I'm just curious as to why there don't seem to be any reports reported and we would expect understandably, there would be some. These are the folks we work with. Doing that in a way that is open and not judgmental.*

				(Participant 11)
	You need to be able to set appropriate performance indicators for YMH services	9/5	*Evidence of strong beliefs impacting on behaviour, relates to a youth mental health service belief that PHNs expectations are unrealistic*	*I think people underestimate how hard it is to develop a really good indicator… You have to be really careful because you can create perverse incentives.* *(Participant 16)*<br><br>*…when you're actually setting a performance against expectations, or expectations of outcomes, it's really defining or ensuring that you understand what those outcomes are and how relevant they are to what you're actually trying to achieve.* *(Participant 20)*
Memory, attention and decision processes	We pay closer attention to commissioned services when we have concerns about them	12/8	*Evidence of strong beliefs impacting on behaviour*	*I think the thing that motivates us to use the data the most is when we have concerns around performance. Then the higher those concerns go, and then the higher the contract escalation process goes, the more attention that data gets.* *(Participant 1)*
Behavioural regulation	Improvements in monitoring and evaluation planning	13/8	*Evidence of strong beliefs impacting on behaviour, belief shared with youth mental health services*	*I would like to have a plan so that we can say that actually we are capturing everything that we need to and we have a really clear idea of how we know what's working for whom and what circumstances.* *(Participant 1)*
	Greater access to data collected by headspace centres	13/7	*Evidence of strong beliefs impacting on behaviour, belief shared with youth mental health services*	*I think the data we do get from Headspace National is really good, it's just that it could improve by providing us with the raw data… so that we can really manipulate the data and evaluate it as we need to* *(Participant 15)*
	Improvements in ability to compare commissioned services	7/5	*Evidence of strong beliefs impacting on behaviour*	*If you looked across all youth services to develop a framework where you could get to some standardised feedback on services that could be put together at a national level.* *(Participant 22)*
	Improvements in data processes and tools	11/7	*Evidence of strong beliefs impacting on behaviour, belief shared with youth mental health services*	*I'd like to see more automation of processes of data collection at the point of service encounter where we use people's time when they're sitting in the waiting room to collect validated instruments or from their family members to make it easier*

				and more efficient for providers to enter that sort of information. *(Participant 17)*
Environmental context and resources	I am able to access staff in my organisation with ME-related skills	18/15	*Frequently mentioned, conflicting beliefs present, belief shared with youth mental health services*	*…the thing that makes it easier is the monitoring an, evaluation reporting officer. We have that skill in the PHN so that makes it easier. If there is something that I - some data I need analysed or whatever, there is that skill within the PHN to help support doing that.* *(Participant 13)* -----*Conflicting belief*-- *I often feel like we've probably don't have as much knowledge and skill and capability in understanding how best to set that in terms of your data collection within something like a PHN organisation. To me there are people that have been doing this for their whole careers and can give us incredibly good advice, but you don't necessarily have that within your organisation.* *(Participant 19)*
	I lack the time to dedicate to monitoring and evaluation	21/12	*Evidence of strong beliefs impacting on behaviour, belief shared with youth mental health services*	*…there's less data analysis than I would like happening. That's often due to time restraints* *(Participant 1)*
	Commissioned services vary in their ME capability	19/11	*Evidence of strong beliefs impacting on behaviour*	*I think some lead agencies are great at what they're doing with data, but some - certain lead agencies, smaller lead agencies, don't know what to do with it either and don't see the benefits of it.* *(Participant 40)*
	It can be difficult to use the data we receive from headspace National Office for monitoring and evaluation	26/11	*Evidence of strong beliefs impacting on behaviour, conflicting beliefs present, belief shared with youth mental health services*	*…we also have access to Tableau, which is the system that collects all of the information that the Headspace teams are putting into their data system. But I find that system a little bit restrictive, because it's not - it's sort of like a visualisation tool as well, it doesn't provide the raw data, so we can't - if we wanted to compare outcomes of the K10, for instance, we can't access the pre- and post-outcome scores. It's just very limited.* *(Participant 15)*

				-----Conflicting belief--- *Then the outcome data, so again the Tableau I find is really accessible, so it's quite nice to be able to jump in and access the data in that sort of form. Particularly looking at some of the outcome data that the headspace spits out.* *(Participant 19)*
	My organisation is supportive of the use of monitoring and evaluation information	15/8	*Conflicting beliefs present, belief shared with youth mental health services*	*…from the Board through our CEO and Executive down have a culture of wanting to use monitoring and evaluation data to drive improvement so it's highly value. I think we have a stable workforce that's fairly data literate. That also understand the value of using data to drive improvement.* *(Participant 17)* *-----Conflicting belief---* *Demonstrating the value of it to people who, historically, haven't really cared about it is hard. It's my job to do that and I need to be better at it. But it's not easy, sometimes, to justify why you need another person in an evaluation team versus another person in a contract management team.* *(Participant 16)*
	The Primary Mental Health Care Minimum Data Set is of limited use for monitoring and evaluating commissioned services	17/8	*Evidence of strong beliefs impacting on behaviour*	*The PMHC MDS is not fit for purpose. It has too many fields. It collects information that we don't necessarily use or value. It creates a reporting burden for provider organisations that's unnecessary and unwarranted. It needs to be reviewed.* *(Participant 17)*
	It feels like headspace centres have two masters	14/6	*Evidence of strong beliefs impacting on behaviour, belief shared with youth mental health services*	*I think the other complexity or the other component in there is you've got services - well the early psychosis or Headspace which have a very heavy national input into that report. So, if you look at hAPI, hAPI is more accessible to Headspace national office than it is to PHNs and local organisations looking at the performance of the Headspace centres. They're just as accountable to Headspace national office really as they are to us.* *(Participant 22)*

	There is limited funding for PHNs to allocate to monitoring and evaluation	8/5	*Evidence of strong beliefs impacting on behaviour*	*What would be a nice incentive is if we got some extra funding that was specific to evaluation. That would be a nice incentive that we don't have… otherwise it's making that choice about, do you use that money for delivering a program which is really necessary, or evaluating a program that already exists.* *(Participant 1)*
	Data processes and tools are problematic	10/5	*Evidence of strong beliefs impacting on behaviour, belief shared with youth mental health sample group*	*We have a database that the PHN manages, which all of the service providers enter into, which collects the MDS so that we can just export an Excel spreadsheet straight from that.*<br>*It does come with some challenges because the service providers often have a lot of difficulty - it's not the best system. It's quite limited in what it can do with reporting. So the service providers often have challenges in being able to export and being able to filter according to the KPIs and how they should be filtering.* *(Participant 3)*
	Politics can undermine the use of ME information in decision-making	10/4	*Evidence of strong beliefs impacting on behaviour*	*The reality is in the Australian commissioning environment the politics of commissioning, the relationships with government, and the proximity of government to their commissioners means that we can make a decision to say, well, the K10s are getting worse.*<br>*You're not achieving any outcome here. But government will say, you will continue to fund them anyway. That makes a bit of a joke of the whole use of analytics to drive improvement process.* *(Participant 17)*
	Commissioned services have limited funds to allocate to monitoring and evaluation	4/4	*Evidence of strong beliefs impacting on behaviour, belief shared with youth mental health services*	*The difficulty is the service providers, I really feel for them, because they live and breathe off the contracts they get. We don't build in specifically into the contracts that evaluation time and resource and that generally speaking they're expected just to do that as part of the work that we commission. But our commissioning dollars are reasonably tight, so when we write the contracts we deliberately write the contracts for the majority of that money to be spent on direct service delivery.* *(Participant 19)*

S2 Appendix. Supp. Results Table

Social influences	I learn from and collaborate with other PHNs	25/14	*Evidence of strong beliefs impacting on behaviour, conflicting beliefs present*	*I actually spoke to four other PHNs to get their data to see what they collected and what some of their turnaround times were, which was fantastic. So we've done our own little benchmark study.*<br>*(Participant 12)*<br>*-----Conflicting belief--*<br>*Researcher: How about other PHNs? Are you influenced by them in any way?*<br>*Interviewee: Not really [laughs]. No. No, not really, no.*<br>*(Participant 13)*
	It's useful to access the support of national youth mental health organisations (e.g. Orygen and headspace National)	12/10	*Evidence of strong beliefs impacting on behaviour, conflicting beliefs present, belief shared with youth mental health services*	*…helping us with the data collection and understanding what it means. I used to go to a lot of the Orygen functions and that really helped in my mind piece together how you design, implement and measure a youth mental health service, which is not just how you measure any kind of service. So, they've been great.*<br>*(Participant 16)*<br>*-----Conflicting belief--*<br>*It was more about me contacting headspace head office to find out information. Like around data, I had to make that call to the data person to be able to be asking them to give me certain information. It feels like they're just the gate keepers. They work at their own - I don't know if they work at their own pace. There's no relationship there with me with any certain person…as a whole, it's been really difficult.*<br>*(Participant 40)*
	The Australian Government Department of Health is supportive but does not always communicate well	8/6	*Evidence of strong beliefs impacting on behaviour*	*…the Department - they're really supportive of us. They - I'm the chair of a national network for the PHNs to do planning. They just - if we find a thing that's not working very well, they'll support it because the want the program to work.*<br>*(Participant 16)*<br>*…there's a number of the people in the Department all working on PHNs and they don't always communicate and there isn't always a consistent message. That's probably the main challenge from the Department.*

				(Participant 16)
	I have supportive relationships with staff in other areas of the PHN	7/6	*Conflicting beliefs present*	*We work together pretty well as a team. Our program coordinators try and link in together, so that we're learning off each other.* *(Participant 2)* -----*Conflicting belief*--- *I am, in an essence, I am quite siloed. I look after certain contracts no one else, except my manager, really has much input.* *(Participant 40)*
Beliefs about capabilities	Monitoring and evaluation may identify an issue which we are unable to support a service with	7/4	*Evidence of strong beliefs impacting on behaviour*	*I think the downside will probably be if I've found a need and I can't support that or I can't move it forward and just shrug my shoulders to say oh it's just the way it is. So if I'm aware of a gap or if I'm aware that someone is struggling and I can't assist, I think that's sort of a negative of evaluation.* *(Participant 34)*
Optimism	The PHN continues to get better at monitoring and evaluation	23/13	*Strong levels of optimism present among many PHNs*	*Researcher: Are you optimistic about the potential for use of information?* *Interviewee: I'd say so. We're getting better, because the PHNs have only been around for a little while and they've only been evaluating, or well, monitoring for a little while. So I think we are getting more skilled and we're getting better templates each time, and we're getting better workforce capacity within the PHNs. Yeah, so we're getting there.* *(Participant 1)*
Intentions	Want to reduce collecting data for the sake of collecting data	10/7	*Evidence of strong beliefs impacting on behaviour*	*I've been really keen to try and reduce collecting data for collecting data's sake. So unless someone can tell me we've collected this data to look at specifically this outcome that we were after and it relates to the program logic in this way, then for me I'd be keen to limit how much we just - we throw out data items just to see what we get back.* *(Participant 19)*
	Monitoring and evaluation is an integral part of the work we do	8/8	*Evidence of strong beliefs impacting on behaviour, belief shared with youth mental health services*	*I mean in terms of benefits, you just can't function without it. Particularly in the whole commissioning kind of way of working.* *(Participant 1)*

	We intend on improving our use of monitoring and evaluation	32/11	*Evidence of strong beliefs impacting on behaviour, belief shared with youth mental health services*	*…there'll be more formal conversations with providers around how we might continue to improve the way that we monitor and evaluate the programs together.* *(Participant 11)*
	We intend on increasing the involvement of young people and families in monitoring and evaluation	7/7	*Evidence of strong beliefs impacting on behaviour*	*Well that's a good question because they will definitely be involved - or people with lived experience will be involved in a codesign piece that we're doing absolutely. So in that way I guess moving forward, they will be involved in how we monitor and evaluate these new services.* *(Participant 37)*
Beliefs about consequences	It helps me to understand what is happening in the service and informs improvement	23/14	*Evidence of strong beliefs impacting on behaviour, belief shared with youth mental health services*	*I think the benefit is you get a more complete picture of what's actually going on, if you're going to collect the data and then analyse it and use it to inform decisions.* *(Participant 4)*
	Qualitative data is needed to contextualise quantitative data	24/11	*Evidence of strong beliefs impacting on behaviour, belief shared with youth mental health services*	*…we receive monthly data and they've got a target and an achievement. Really they're only numbers on paper, until you understand what they actually mean. So I find that the qualitative stuff behind the data is real - it's of equal importance, because it speaks to the data. I think that tells us the richest information.* *(Participant 24)*
	It is beneficial to engage with services on an ongoing basis	9/9	*Evidence of strong beliefs impacting on behaviour*	*There's probably a lot more discussion that happens on those brief phone calls and those different meetings that you're having throughout the month where you're probably discussing more about what's happening on the ground, what does this mean, what are you seeing.* *(Participant 20)*
	Monitoring and evaluation is burdensome for service providers	14/9	*Evidence of strong beliefs impacting on behaviour, belief shared with youth mental health services*	*The burden of sometimes collecting that information is relatively large, because firstly you have to determine what information that is, and secondly collecting it is often - MDS largely comes from clinical notes and records, so you have to do - you tend to be doing a lot of that information anyway, from a governance perspective, when you're keeping notes that you need to. The other information is the stuff that's not so easily accessible.*

				(Participant 22)
	Knowing what is going on informs how we support services	20/8	*Evidence of strong beliefs impacting on behaviour*	*Researcher: are there any benefits and downsides to not using monitoring and evaluation information?<br>Interviewee: Yeah, you're just not capturing info. You can't advocate for a program, if you're seeing it working well or not working well. You - I guess, as a funding agency, we can't look at other strategies, or other things implement like, well, yeah. Yep. So many disadvantages not to having it.<br>(Participant 2)*
	The data does not always accurately reflect what is happening on the ground	21/8	*Evidence of strong beliefs impacting on behaviour, belief shared with youth mental health services*	*What we're finding is we're slowly pulling the data sets together, particularly where often, not always, but often the provider's local data set shows a more positive story than what our PMHC MDS data set shows as a point of truth.<br>(Participant 19)*
	It is difficult to make comparisons between services	10/7	*Evidence of strong beliefs impacting on behaviour*	*You pitch one headspace service against another. You're talking about different demographics, different accessibility. Your talking - there's a lot of different elements. How do you - what do pitch against the national average? Headspace in a metro area versus headspace in a rural area. They're going to be different.<br>(Participant 40)*
	It helps with identifying service issues (including risks and gaps)	6/6	*Evidence of strong beliefs impacting on behaviour, belief shared with youth mental health services*	*…because of that regular consideration of the progress reports, I knew exactly what the weak points were for each centre<br>(Participant 38)*
Goals	Monitoring and evaluating helps to ensure young people receive the best care possible and experience improved outcomes	28/14	*Evidence of strong beliefs impacting on behaviour, belief shared with youth mental health services*	*…ensuring that we're providing the right service for the young person, the actual user of this service. I think that's the biggest incentive of all and that is why we're all doing our jobs.<br>(Participant 12)*
	I want to understand the experiences of young people and families	10/8	*Evidence of strong beliefs impacting on behaviour, belief shared with youth mental health services*	*…getting the young persons' response as well is probably more so important. Basically, the end users' response is what matters because essentially that's what the service is being commissioned to achieve.<br>(Participant 20)*

	Attending to other parts of my job may delay ME but does not prevent me from doing it	10/8	*Evidence of strong beliefs impacting on behaviour*	*Researcher: Is there anything which takes higher priority than the monitoring and evaluation of these programs? Interviewee: On a day-to-day basis, there might be. But over the course of the year, that's my job.* *(Participant 16)*
	I want to develop a partnership approach to working with services	13/8	*Evidence of strong beliefs impacting on behaviour*	*I'd like to be able to see us over time have a relationship where if they want to know more and they want some analysis about something that they can come to us and we can support them in that.* *(Participant 17)*
	I want to support services with quality improvement	11/8	*Evidence of strong beliefs impacting on behaviour*	*We can support those services and identify what the main issues are, then, yes, encourage them to improve what they're doing.* *(Participant 15)*
	Monitoring and evaluation should inform quality improvement	10/5	*Evidence of strong beliefs impacting on behaviour, belief shared with youth mental health services*	*Try to use it to drive improvement rather than it being something for performance management. We've had an intentional focus on data for driving improvement in how we deliver care and not using it to beat people up.* *(Participant 17)*
Reinforcement	The Australian government's expectations of PHN performance significantly influences what data PHNs ask services to collect	15/10	*Evidence of strong beliefs impacting on behaviour*	*…initially, we had services record against an outcomes framework… As the focus became more on the PMHC-MDS from a government perspective, the call was made to stop doing all that other recording; they only need to do PMHC-MDS.* *(Participant 22)*
	The Australian government's expectations incentivises a focuses on service activity rather than service outcomes	11/5	*Evidence of strong beliefs impacting on behaviour*	*…the Department look at the wrong data. They look at outputs. They kind of have to, but also, they kind of shouldn't. I see that they're in a hard position, but it should be all about the outcomes.* *(Participant 16)* *…the easiest one to look at, information to look at, is just the outputs, though I don't think that's very helpful. So I think that generally, the PHN data - the PHNs generally do look too much to outputs* *(Participant 13)*

S2 Appendix. Supp. Results Table

**Youth mental health services**				
Skills	You need to be data literate	26/7	*Belief shared with PHNs, evidence of strong beliefs impacting on behaviour*	*…having a background knowledge in statistics is helpful, particularly if we are looking at some of those other databases or systems that we keep. If we want to extract information from those it's definitely helpful to have that background (Participant 14)*
	You need to be an effective communicator	16/11	*Evidence of strong beliefs impacting on behaviour*	*I think you need to be a good communicator. I think that people who -there are some people who can see data and they can talk data, there are some people who can talk young people and mental health and I think you've got to have both those skills. You've got to be able to look at data and then interpret it for your audience. (Participant 10)*
	You need to be able to empathise with YMH service staff	17/10	*Evidence of strong beliefs impacting on behaviour, belief shared with PHNs*	*…empathetic towards colleagues. So you kind of understand people's mindset and so if they're not doing so something, if they're not achieving something, it's not because they don't give a fuck. It's because there's something going on with the client, or with them or whatever. Very rarely would you see someone in a job like this who is just being lazy, so you kind of have the mindset of I guess (Participant 27)*
	You need a good understanding of evaluation	13/8	*Evidence of strong beliefs impacting on behaviour*	*Understanding how to evaluate and how to develop an evaluation project is really important. (Participant 29)*
	You need a good understanding of the YMH service context	9/6	*Evidence of strong beliefs impacting on behaviour, belief shared with PHNs*	*I have done direct work with young people so I am very connected to the operational side and I understand what that's like. It's easier to interpret what's happening when you know what it means on the ground. It's easier to make changes. (Participant 10)*
	You need to be able to apply the data to the real-world	6/6	*Evidence of strong beliefs impacting on behaviour*	*I guess it's about being able to understand, what does that data mean for us as a service? It's one thing to understand this is what the data is telling us, but how do we also - what's the application of that in our day-to-day work, and what are the practical implications of that? (Participant 14)*

	You need a good understanding of data processes and tools	5/4	*Evidence of strong beliefs impacting on behaviour*	*I need to know what hAPI does and I need to know what RediCASE does, because if I don't know how the data reporting system works, where people input what for me to pull that thing out, then it means nothing to me. That training is really important as well.* *(Participant 10)*
	You need to be able to build relationships with other organisations	7/5	*Evidence of strong beliefs impacting on behaviour, belief shared with PHNs*	*Oh I think definitely engaging with the PHN and with stakeholders. I think building a good relationship with the PHN is really important and also being able to build a good relationship with the LHD services, so that's the public health services.* *(Participant 31)*
	You need to be inquisitive and open minded	8/4	*Evidence of strong beliefs impacting on behaviour, belief shared with PHNs*	*If you're open to something, being okay if something's not working as you expected, then it's okay. But if you don't really want to know that, then I can see that being a downside to evaluation.* *(Participant 32)*
Behavioural regulation	The rationale for data collection needs to be meaningful to staff	14/9	*Evidence of strong beliefs impacting on behaviour*	*If people have a good understanding of why – also outcome measures aren't just created to fulfil just financial gain or funding all the time, it's usually in good intentions for the consumers and families and to improve service delivery. So I think if staff have a good understanding of that they're more willing to adapt as well.* *(Participant 28)*
	Improvements in monitoring and evaluation planning	13/9	*Evidence of strong beliefs impacting on behaviour, belief shared with PHNs*	*We could do with a little bit more guidance on what they would like us to collect. I think we've had three different version of the work plan now and each time it has shrunk, but I do look at it and I know that they're adapting a work plan from big business and that it doesn't quite fit what we're doing. I think that, yeah, having a clearer idea of what they would like reported on, how they'd like it reported, would have been really helpful.* *(Participant 29)*
	Improvements in data processes and tools	17/8	*Evidence of strong beliefs impacting on behaviour, belief shared with PHNs*	*The HAPI and Tableau is one thing, EMR is another thing, the stuff that the PHN sometimes want, that could be a different occasion of service type thing. Mainly it's between our EMR*

				and HAPI, are the two main ones. If there was some way we could – this is a pipedream but if we could get some place that could pull the data automatically from that system and this system then that would be good. *(Participant 8)*
	Improvements in feedback provided by PHN	11/7	*Evidence of strong beliefs impacting on behaviour*	*Yeah, well, I think getting [feedback], for a starter, would be great, but - and getting it a contextualised way from someone who kind of understands how to present that information.* *(Participant 6)*
	Create formal opportunities to discuss data with staff	6/5	*Evidence of strong beliefs impacting on behaviour*	*All of our data collection wasn't - it wasn't informing how we practice. So again, as part of our whole quality improvement process, we folding that into the case review and making that a little bit more informed by data.* *(Participant 35)*
	Monitoring and evaluation should be adequately resourced	6/5	*Evidence of strong beliefs impacting on behaviour*	*Having positions in place that can support teams in performing to that level as well. Because otherwise it feels like you're trying – you're probably stretching trying to do too many – fulfill too many roles at once. That's where you do I think fall down with again losing sight of what you're trying to achieve and the outcome measures versus trying to fulfill clinical positions. It can be quite tricky.* *(Participant 28)*
	Decisions about data collected should be made collaboratively	13/5	*Evidence of strong beliefs impacting on behaviour*	*…what I'm getting at is like anything if you're expecting good results it should be a collaboration. There should be openness from both sides. So the - I would say what I want to know is are we seeing a lot of people and are they getting better. The PHN might also say, look are they getting jobs or something. Fine. That's reasonable. So it should be a collaboration between the commissioning agent and the service and young people and families in the conversation. What do they want to be measured?* *(Participant 9)*
	PHN reporting requirements should be streamlined	6/4	*Evidence of strong beliefs impacting on behaviour*	*I find that sometimes there can be - we're submitting multiple reports sometimes with the same information, or if there was perhaps a way - I know that different contracts will need different reports at different intervals, but perhaps if there was*

				a way that it could be a bit more comprehensive, or we're not having to provide that same information again, or duplicating information.<br>*(Participant 14)*
	Greater access to data collected by headspace centres	7/3	*Evidence of strong beliefs impacting on behaviour, belief shared with PHNs*	*Researcher: Say you were able to get the raw data out of hAPI, how would that - what sorts of things would that help you to do, or what parts of your job would be easier?*<br>*Interviewee: Well if I was able to query it, then it would save me having to fill out this little database. I mean that's just silly. What I'm doing is silly, I know that. But yeah, or if they allowed me to use Tableau to get the information out myself…*<br>*(Participant 27)*
Environmental context and resources	I lack the time to dedicate to monitoring and evaluation	25/15	*Belief shared with PHNs*	*…its' really time consuming and it's often done after hours.*<br>*(Participant 29)*
	Data processes and tools are problematic	51/15	*Evidence of strong beliefs impacting on behaviour, conflict beliefs present, belief shared with PHNs*	*…there's been lots of confusion because we have an internal database and then there's the PHN database and they don't communicate because we're an NGO with no money, community managed, so our database, I think, could be a lot better but it's not.*<br>*(Participant 18)*<br>*-----Conflicting belief--*<br>*The hAPI platform itself can actually generate reports or collect a lot of that information. Particularly the new system is quite sophisticated in the sense that it can just extract an actual report for you in real time.*<br>*(Participant 14)*
	My organisation is supportive of the use of monitoring and evaluation information	13/12	*Evidence of strong beliefs impacting on behaviour, belief shared with PHNs*	*I think also their commitment as well to the data and the monitoring and evaluation, like it is something they really hold to a high standard. So they want the information, the board wants the information and they want to understand it as well and it is a collaborative thing, like it's a two-way conversation sometimes. I think that's really helpful.*<br>*(Participant 10)*
	I am able to access staff in my organisation with	17/8	*Evidence of strong beliefs impacting on behaviour,*	*I was really lucky that we had a person who had done a significant amount of evaluation and has also – there was*

	monitoring and evaluation-related skills		*conflict beliefs present, belief shared with PHNs*	*another person that had also been involved in teaching medical students how to do their end-of-year projects. So I was lucky. They'd just say, this is what you need to do, this is how you set it up.* *(Participant 29)* -----*Conflicting belief*--- *We do have staff that - they're not that familiar with data and they haven't used lots of different ranges of IT systems.* *(Participant 28)*
	It feels like headspace centres have two masters	21/6	*Belief shared with PHNs*	*They [the PHN] inherited both our Headspace Primary and the hYEPP. That was in existence before. My understanding is they can't take money out of it. I'd be very confident that our PHN would have not chosen the measurements that are done by Headspace National. I really feel for the PHN. I think - so I'm not critical - I don't think they have any power. Except they get put in a difficult position because what they're doing is - all they're doing is monitoring our compliance with something they have no control over, which puts at risk our relationship.* *(Participant 9)*
	Commissioned services have limited funds to allocate to monitoring and evaluation	7/5	*Belief shared with PHNs*	*We did actually, I think, originally request some quality and safety funding to go into our budget, which, in our organisation, would capture things like data and evaluation, but that was not supported, because it was very much client services, staff on the ground, versus the support to the program was not funded.* *(Participant 6)*
	It can be difficult to use the data we receive from headspace National Office for monitoring and evaluation	7/3	*Evidence of strong beliefs impacting on behaviour, conflict beliefs present, belief shared with PHNs*	*We actually don't have an enormous amount of use for the information that comes back from [Headspace] head office, partly because the information's de-identified. In the process, even though the information comes from our service and from data that we input, they believe that they're restricted in terms of giving that information back to us. So they'll give us back information, but it's only according to client number, not according to the client's name and that creates a lot of problems for us in terms of working out basically, in terms of using the information essentially for service improvement.*

				(Participant 27)<br>*-----Conflicting belief---*<br>*Then every quarter that information is collected and provided back to the service about how many sessions, how many young people were seen and what were they presenting with, what was their age. So, we do get a lot of information from that platform about the young people and the demographics and I guess the story of our headspace centre and primary. I find that really helpful.*<br>*(Participant 26)*
Social influences	The PHN actively supports us	34/14	*Evidence of strong beliefs impacting on behaviour, conflicting beliefs present*	*…the PHNs that I find helpful are the ones who are willing to work in partnership, rather than seeing it as a - there are commissioners who have described themselves as like an ATM, you complete the transaction and we give you the money. Whereas others are more likely to work in partnership, so really collaborative kind of decision making.*<br>*(Participant 31)*<br>*-----Conflicting belief---*<br>*What I found very, very difficult with them [the PHN] is their just obsessiveness with the bureaucracy of the data collection. Just the - is your I dotted, is your T crossed, you know? It was just really way over the top and not using the data that effectively.*<br>*(Participant 39)*
	Staff within my service value the use of data differently	14/9	*Evidence of strong beliefs impacting on behaviour*	*harping on about best practice and evaluation might be experienced as threatening for some staff whose confidence in their practice may be different or their appreciation for monitoring may be different. But for example, there is at least a couple of individuals who feel strongly against progress monitoring because from a practitioner, philosophical point of view they conclude that that inhibits rather than helps.*<br>*(Participant 33)*
	I learn from and collaborate with other youth mental health services	21/8	*Evidence of strong beliefs impacting on behaviour*	*Perhaps comparing ourselves with other centres, we do keep in frequent communication with the other centres in our region as well. We may use that as a bit of a guide for not necessarily*

				benchmarking, but any quality improvement or themes or trends as well.<br>*(Participant 14)*
	It's useful to access the support of national youth mental health organisations (e.g. Orygen and headspace National)	10/8	*Evidence of strong beliefs impacting on behaviour, belief shared with PHNs, conflicting beliefs present*	*I think Orygen are actually really good at doing this when they come out on fidelity visits – is we're trying to achieve – the service delivery and the KPIs and stuff that are set, it's for the benefit of the consumers and families.*<br>*(Participant 28)*<br>*hNO. If I don't understand anything or want to interpret something - I am not a researcher by background, so I do look to hNO for that kind of direction and support.*<br>*(Participant 30)*<br>*-----Conflicting belief---*<br>*Interviewee: I think everyone's just gone into their shells a little bit in those roles.*<br>*Researcher: have you got a sense of why that is?*<br>*Interviewee: People feel that they're not listened to. That's a pretty big part of it and to some degree I think not very well regarded I don't think, by some of the hNO people that are around now, that people in our roles aren't very highly regarded. You pick stuff up like that.*<br>*(Participant 27)*
	I have a supportive manager	8/6	*Evidence of strong beliefs impacting on behaviour*	*The Clinical Director for instance is an ideas man. So he looks at the data and he thinks about all of these different ideas, whereas I look at the data and I analyse, look at how we're meeting our KPIs and what we need to change. Whereas he then just takes it on a whole other level which is great, because I guess yeah, we work really nicely together and we kind of complement each other. So that's probably - I'm learning from the Clinical Director as I go, as we have these conversations.*<br>*(Participant 31)*
Beliefs about capabilities	My monitoring and evaluation skills could be better	19/9	*Evidence of strong beliefs impacting on behaviour, conflicting beliefs present*	*Researcher: You're pooling raw data and then you're doing some analysis, is that right?*<br>*Interviewee: Yeah, which is I think, as a clinician and not having had specific training in data collection and data*

				analysis and have not really had a lot – been involved with, but not had a lot to do with evaluation of services or stuff like that, that's been quite a challenge. The first couple of reports we probably didn't quite get that right in terms of what the PHN wanted and I think it's still a work in progress. *(Participant 29)* -----*Conflicting belief*--- *I feel like I know how to interpret and I suppose, implement changes et cetera from the data we collect here. I understand it really, really well.* *(Participant 25)*
Emotion	I worry about the potential consequences of not meeting the PHN's expectations	12/5	*Strong emotions present*	*It can also make me feel nervous. I guess I had a lot of anxiety when we'd had to do the Q3 report when I'd first started and I had to put zero next to a lot of our KPIs. That was very anxiety provoking.* *(Participant 10)*<br><br>*My concern is what happens at the end of your contract, because that's probably the final answer on whether your data was of benefit or not.* *(Participant 6)*
Intentions	Monitoring and evaluation is an integral part of the work we do	12/9	*Evidence of strong beliefs impacting on behaviour, belief shared with PHNs*	*You can't do direct service work without having the numbers and the information, evaluation stuff behind it* *(Participant 30)*
	We intend on improving our use of monitoring and evaluation	14/7	*Evidence of strong beliefs impacting on behaviour, belief shared with PHNs*	*We have started to have conversations around actually developing our own monitoring and evaluation frameworks that look at – add to those contractual obligations and look at what do we think is important to monitor and how often.* *(Participant 32)*
Beliefs about consequences	Monitoring and evaluation is burdensome for services	26/13	*Evidence of strong beliefs impacting on behaviour, belief shared with PHNs*	*The amount of information that's collected is huge. It actually makes - impacts on direct client care, so you're just needing to balance that out.* *(Participant 30)*
	It helps me to understand what is happening in the	37/13	*Evidence of strong beliefs impacting on behaviour, belief shared with PHNs*	*I think realistically data does - the positives outweigh because it just gives me such a huge wealth of information about what's happening and what we need to do differently.*

	service and informs improvement			*(Participant 10)*
	The data we collect informs clinical practice	29/12	*Evidence of strong beliefs impacting on behaviour, conflicting beliefs present*	*…it can be somewhat meaningful when we're asking the staff to be administering like the K10 plus the SDQ, that they're able to actually use some of that in their therapeutic interventions to understand where clients are at, which in a lot of cases you can do that.*<br>*(Participant 35)*<br><br>*Interviewee: it's challenged people with COVID but that's been a good challenge, actually.*<br>*Researcher: Yeah?*<br>*Interviewee: It's really - well we never had in our procedure manual at all about telephone counselling… and we know from the data that a lot of young people like that. So it's very interesting that clinicians are drifting back to face to face, whereas we're able to use the data to say 'well actually, make sure you offer a choice because the data shows us that young people might actually prefer telephone than face to face.'*<br>*(Participant 39)*<br><br>*----Conflicting beliefs---*<br>*So if you are asking clinicians to spend a number of sessions asking a whole lot of problem saturated symptom type questions to work out exactly what's wrong with someone, then in fact you're not having an open mind and listening carefully to what people's perspective on the best way they can get out of their situation and helping them with that, they're very counter approaches.*<br>*(Participant 9)*<br><br>*…if they tell you, use this tool. You go yes, and again what we found the impact that makes on the clinicians on the ground is that again they just think I need to do this with 90 per cent of my clients.*

				They don't sort of think 'actually what do I do with that information?' I think it's really easy for them to lose the purpose of actually why was this tool developed in the first place… We just found, yeah, they were doing it for the sake of doing it and not feeding that information back to people. *(Participant 32)*
	The PHN provides little feedback on the data we provide them with	17/9	*Evidence of strong beliefs impacting on behaviour*	*As far as evaluation, in my experience there isn't a lot of communication back as far as if there has been any issues raised within the reporting period. It can be a difficult space at times to get that feedback back from the PHN.* *(Participant 26)*
	Monitoring and evaluation helps to ensure we operate in an evidence-based way	9/8	*Evidence of strong beliefs impacting on behaviour*	*Without evaluation and reflection and looking at ourselves and looking at what we're doing, we could be in the dark ages. We could be providing a service that is unhelpful but because of, I don't know, ego with their 'oh no, I do the best here; we do fine'. It means that we can't not be focused on outcomes in the participant and their needs and keeps us ethical and keeps us up-to-date with best practice.* *(Participant 33)*<br><br>*…but as a really good clinician and as someone who works in psychology, social work, whatever, there still needs to be some sort of scientific basis. We still need to be using some sort of evidence, so it needs to be evidence-based practice.*<br>*Our screening tool, our eligibility criteria, ties into our evaluation methods. All of our ongoing determining eligibility for young people, even a year in the program, ties into our evaluation methods.* *(Participant 35)*
	It helps to identify service issues (including risks and gaps)	10/8	*Evidence of strong beliefs impacting on behaviour, belief shared with PHNs*	*Looking at the gaps of who you're not servicing, and that's been one of the big things around the data and going, okay, maybe [deidentified geographic area] has a big area of Indigenous young people. We haven't really - we're actually a lot better than we were at the start, and going, okay, what*

				links do we need to make in order to be more present and available to different cohorts of young people? *(Participant 7)*
	Qualitative data is needed to contextualise quantitative data	12/7	*Evidence of strong beliefs impacting on behaviour, belief shared with PHNs*	*I think it's really the case studies that are particularly useful, because we can really get a good sense, ourselves, around what the presentations were for young people, what their goals were, what our evidence-based approaches were to meeting those goals, where the young person came to in their trajectory, and what the outcomes were for good, for bad, for otherwise, and also what the service impacts have been within service.* *(Participant 6)*
	The data does not always accurately reflect what's happening on the ground	21/7	*Evidence of strong beliefs impacting on behaviour, belief shared with PHNs*	*I don't think that any of those measures should be taken individually. I think that would be reductionistic... they all need to be collected and viewed as a whole. I think to take any one of them individually and use that as the basis for the outcome is totally not valid.* *(Participant 8)*<br><br>*The data that the MDS captures, which informs the majority of our episodic data and our client presentation, really is not youth-friendly at all, and it's very medical model structured. So, that, for me, is the biggest challenge that we have. That's where most of our data is captured, and that, I think, is the most inaccurate representation of what we're doing.* *(Participant 6)*
	Providing feedback to staff helps to boost morale	8/4	*Evidence of strong beliefs impacting on behaviour*	*I think it's also really important for me to reflect some of that data back to the team, because I truly believe that they work really, really hard.*<br>*They do a really good job, so what I also like to do is at the end of each reporting period, provide a little summary to the seniors, so they can share with the teams some of the work that they've been doing, any sorts of increases, trends, some good news stories that they've shared with me, to be able to I guess reflect that back to the team, so they can actually see that their hard work isn't going unnoticed.*

				(Participant 31)
	There are issues with how we elicit feedback from young people	7/4	*Evidence of strong beliefs impacting on behaviour*	*The problem with the family and friends surveys in HAPI is that if it's being done by clinicians, it's like literally here you go, here's the computer or here's the piece of paper, you just mark off what you think of me and the service. I will then enter that into our system and be able to see that all the time. It's not anonymous. It's not – it's just awkward.* *(Participant 8)*
Goals	I want to understand the experiences of young people and families	22/13	*Evidence of strong beliefs impacting on behaviour, belief shared with PHN*	*I'm very interested to see what the young person was experiencing when they start with you versus what they're experiencing at the end of an episode of care. So there is a bit of a personal incentive to see, well have we made a difference, have they appreciated it, have we done the right thing, what could we do better?* *(Participant 29)*
	Monitoring and evaluation often has to take a backseat to other priorities	18/12	*Evidence of strong beliefs impacting on behaviour*	*Data is a tiny, tiny little part of the operations here and that's just something I accept. It's a service. It's very much a client-oriented, focussed service and so anything that's happening to do with the service or to do with the young person is always, always going to take priority over the sort of stuff that I do.* *(Participant 27)*
	Monitoring and evaluation helps to ensure young people receive the best care possible and experience improved outcomes	17/12	*Evidence of strong beliefs impacting on behaviour, belief shared with PHNs*	*…the incentive is when you have been able to actually look at something and then make a change for the better, in your service, that has an impact and a meaning for the client and for the service.* *(Participant 6)*
	I want to be able to demonstrate the difference the service makes	14/9	*Evidence of strong beliefs impacting on behaviour*	*We want to show that we believe what we're doing is making a difference, our reputation and all the feedback that we get is so positive, that we want to be able to capture that, if we can.* *(Participant 18)*
	Monitoring and evaluation should inform quality improvement	9/7	*Evidence of strong beliefs impacting on behaviour, belief shared with PHNs*	*The data that I'm getting, or the information I'm getting from clinicians to improve service is my priority area, to make sure that, yeah, things run smoothly.* *(Participant 5)*

	The data we collect should be clinically meaningful	13/4	*Evidence of strong beliefs impacting on behaviour*	*…if we were using measures that actually helped people in the room in real time, that it was useful to the patient, that's the number one thing that's important by far. If any of the measures used are not doing that I'm not really sure why we're using them. Because where it's like - we're a clinical service. Our job is to help people. If the measures are not assisting us in that process we shouldn't be using them.* *(Participant 9)*
Reinforcement	Doing monitoring and evaluation helps to maintain our PHN funding	16/7	*Evidence of strong beliefs impacting on behaviour*	*We'd probably lose our funding. [Laughs] I think if we just didn't do it, we won't meet our contractual obligations and that's that. That's our program.* *(Participant 31)*
	The PHN's expectations of the service are unrealistic	16/7	*Evidence of strong beliefs impacting on behaviour*	*Having recently reviewed what their expected KPIs are, I don't feel super optimistic about it, because I don't think they're necessarily realistic what they're asking us to do. My concern would be that we're going to be told we're not meeting an outcome that for me is not that relevant.* *(Participant 10)*<br><br>*…they've made the KPIs based on the idea that you're doing all these brief interventions, and you can't, because the young person hasn't had their needs met. So the KPIs ultimately are not really significant - they're not reflective of the complexity of the young people who are working there* *(Participant 7)*
