# Supplementary material for: ‘More than just numbers on a page?’ A qualitative exploration of the use of data collection and feedback in youth mental health services
Source: PLoS One. 2022 Jul 20;17(7):e0271023. doi: 10.1371/journal.pone.0271023 (PMC9299353; doi:10.1371/journal.pone.0271023)
Supplement: Attachment — Submitted filename: Response to Reviewers.docx [file pone.0271023.s003.docx]

1 May 2022

Dear Dr Scott,

Thank you for the opportunity to revise and resubmit this paper: *‘More than just numbers on a page?’ A qualitative exploration of the use of data collection and feedback in youth mental health services.*

We have amended the manuscript and have provided responses to the reviewers’ comments in the table following. Please do not hesitate to inform me if any further revisions are required.

For any further information, I am contactable by email at [craig.hamilton@orygen.org.au](mailto:craig.hamilton@orygen.org.au)

We appreciate the time taken to review the manuscript.

Yours sincerely,

Craig Hamilton (on behalf of the authors)

| Reviewer 1 Comments | Response to Reviewer |
| --- | --- |
| This is an interesting and well written manuscript on a topic that is important to ensure the sustainability of services. The conclusions drawn are appropriate based on the data presented and appropriate recommendations have been made. I thought the results were particularly will presented, highlighting the domains of the TDF. Throughout the manuscript authors should make explicit which behaviour is being performed and by whom. | Thank you for your comments. We appreciate the review and the constructive feedback which we believe will strengthen the paper. |
| Abstract  1. Design – ‘data was’ should be ‘date were’. Please check the full manuscript for this error | Thank you for noting this. We have amended the manuscript accordingly. |
| Abstract  2. Results – it’s unclear what ‘behaviour’ refers to here and what the ten types of behaviour are. How does this link to TDF? | We have amended the abstract results to include some examples of the sub-behaviours identified in the study. |
| Abstract  3. Conclusions – Could the conclusions be better presented than a list? Makes it difficult to get a sense of impact. | We have amended the abstract conclusion to be more descriptive. |
| Introduction  4. Good and concise overview of the evaluation and feedback. | Thank you for your observation. |
| Introduction  5. It is highlighted there are two main gaps 1) to understand more about how data is used, and 2) barriers/enablers for improvement based on data collected. But the aims for the manuscript do not align with these and instead look to give an account of how data is collected and the barriers to collecting data from these services. Could authors please update this. | Thank you for noting this. We have made amendments to the last paragraph of the introduction (page 5) so that the gaps 1) little is known about current ME practice, and 2) the barriers and enablers to it use, are more clearly addressed in the study aims. |
| Methods 6. Within the study context – could authors please state what type of data is collected (clinical scores, wellbeing, engagement, etc.)? | To illustrate the type of data collected we have included the following text in paragraph two of the introduction: ‘(e.g. program activity, patient mental health outcomes)’. |
| Methods  7. The authors briefly discuss the use of zoom for most interviews. Could you please reflect on how this influenced data collection, e.g., were there any distractions for participants (such as shared office) that may have influenced answers? See for instance the below papers that examined benefits and drawbacks of Zoom:  Oliffe, J. L., Kelly, M. T., Gonzalez Montaner, G., & Yu Ko, W. F. (2021). Zoom interviews: benefits and concessions. International Journal of Qualitative Methods, 20, 16094069211053522.  Archibald, M. M., Ambagtsheer, R. C., Casey, M. G., & Lawless, M. (2019). Using zoom videoconferencing for qualitative data collection: perceptions and experiences of researchers and participants. International journal of qualitative methods, 18, 1609406919874596. | We have included additional information about participants’ context during Zoom interviews and reflections on internet connectivity issues (or lack thereof) on page 6. |
| Methods  8. Could the authors please discuss whether they reached saturation / sufficient information power? e.g., whether they felt that saturation had been reached, they had reached sufficient information power, or whether they were time constrained? | We have included text about reaching data saturation on page 7. |
| Results  9. Throughout the results section could authors please state the number of participants rather than using ‘few’, ‘many’, ‘some’, ‘most’, etc. | Thanks for the comment. In accordance with qualitative methodology, we have chosen not to state the numbers of participants in order to avoid misrepresenting prevalence and instead present qualitative data to provide sense making and rich description. |
| Results  10. Could authors please make clear what the 27 shared belief statements are- either through a clear description or use of a table/figure for example. | On page 13 we have replaced the figure with a table summarising all the shared belief statements. We also erroneously reported that there were 27 shared belief statements rather than 26 – we have amended accordingly. |
| Results  11. There are only nine supporting quotes presented intext and while these are representative there is more scope to include additional data to provide further meaning behind each belief. | We have added four quotes to the results section and in the first paragraph on page 12 we have amended the text to make it more obvious that further illustrative quotes can be found in appendix 2. |
| Discussion  12. Authors mention ‘a complex set of behaviours’ performed. Could authors please make explicitly clear which behaviours are being referred to. | We have amended the first paragraph of the discussion (pages 19-20) to include the 10 behaviours identified in the study. |
| Discussion  13. Authors reflect on their use of the TDF across the strengths and limitations, implications for future research, and conclusions sections. This makes the discussion of the TDF feel fragmented and would be best to have a central discussion within in the main section. The TDF highlights important conclusions and could be discussed in greater depth. | To reduce fragmentation, we have concentrated discussion of the TDF in the strengths and limitations section (pages 22-23). |
| Appendix  14. Could authors please check appendix 2 table to ensure the illustrative quotes are representative of the specific beliefs. For example, Skills domain – Specific belief: You need to be data literate. I do not feel the quote is representative and instead reflects collaboration and belief about capability. | Thank you for noting this error. The Skills domain on page 1 of appendix 2 has been updated with a relevant representative quote. |
| Appendix  15. Could authors please check for syntax errors within the table (use of quotation marks, indenting text, missing letters from beginning of words). | Thanks for the observation. Syntax errors have been addressed in appendix 2. |

| Reviewer 2 Comments | Response to Reviewer |
| --- | --- |
| This manuscript presents the results of a study of a qualitative study of beliefs about monitoring and evaluation (ME) from mental health commissioners’ and staff associated with youth mental health services in Australia. Forty separate 40-70-minute semi-structured interviews were conducted with participants recruited from a pool of 240+ potential participants using the authors’ social network and a snowball approach (asking first-level contacts to forward the invitation to others). A strength of the study is the use of a theory-based approach to developing the qualitative coding scheme using the Theoretical Domains Framework (TDF) to organize the a priori categorization of themes arising from the interviews. Study findings have the potential to further our understanding of ME in youth mental health services both in terms of use and implementation. However, there were several significant limitations that would need to be addressed to improve the scientific rigor and allow readers to better assess the implications of the findings. | Thank you for your comments. We appreciate you taking the time to review the manuscript and the constructive feedback you have provided. We believe addressing them will strengthen the paper. |
| First, the introduction mixes very separate conceptual areas by combining into a single construct the notions of routine outcome monitoring, audit and feedback, and monitoring and evaluation. While there is some overlap amongst these strategies for informing patient care and quality improvement efforts, they are different. Routine outcome monitoring, for example, has a single reference for a 2019 systematic review (6). There are dozens of studies and several systemic reviews and meta-analyses on this evidence-based practice available (e.g., de Jong et al., 2021, see reference below). The utility of this manuscript could be much clearer by focusing on ME and operationalizing what it is, how it is used, and what the known barriers are in the introduction. | We have amended the text in the introduction (including a definition of ME) on pages 4-5 to emphasise that the study’s focus is ME. |
| Second, the methods description is not sufficiently rigorous for a qualitative study of high quality. For example, it is difficult to assess the quality of the recruitment process from what is described here.  Was a single email sent? How does this differ from a sample of convenience? How were additional recruits vetted from the snowball sampling?  In addition, a serious limitation is the lack of description of qualitative methods outside of the TDF coding scheme. What is meant by inductive and deductive coding and how did this arise from pre-planning coding schemes versus being developed as themes arose?  How was quality maintained on the coding itself (e.g., common methods are consensus coding, use of training for inter-rater reliability, etc.). This reviewer is unclear what was meant by the three criteria described in paragraph 3 on page 6 – why these criteria, how and when were they applied, etc.  Without further description, it is impossible for this reviewer to assess the quality of the study, nor to assess the interpretation of findings. In other words, there is little to no description of the analytic approach and methods. | Thanks for your comments. We have amended the methods section (pages 5-7) accordingly. Changes include:   - Inclusion of flowchart and additional text to elaborate on recruitment process - Addition of text to provide more description of the stages of analysis - Addition of text to provide more information about how steps were taken to ensure quality of analysis - Addition of text to describe why and when the three criteria were applied to determine relevance of the TDF domains |
